# Structure and Effects of Cyanobacterial Lipopolysaccharides

**DOI:** 10.3390/md13074217

**Published:** 2015-07-07

**Authors:** Prasannavenkatesh Durai, Maria Batool, Sangdun Choi

**Affiliations:** Department of Molecular Science and Technology, Ajou University, Suwon 443-749, Korea; E-Mails: prasanna@ajou.ac.kr (P.D.); mariabatool.28@gmail.com (M.B.)

**Keywords:** LPS, endotoxin, cyanobacteria, cyanotoxin, TLR, lipid A, sepsis

## Abstract

Lipopolysaccharide (LPS) is a component of the outer membrane of mainly Gram-negative bacteria and cyanobacteria. The LPS molecules from marine and terrestrial bacteria show structural variations, even among strains within the same species living in the same environment. Cyanobacterial LPS has a unique structure, since it lacks heptose and 3-deoxy-d-*manno*-octulosonic acid (also known as keto-deoxyoctulosonate (KDO)), which are present in the core region of common Gram-negative LPS. In addition, the cyanobacterial lipid A region lacks phosphates and contains odd-chain hydroxylated fatty acids. While the role of Gram-negative lipid A in the regulation of the innate immune response through Toll-like Receptor (TLR) 4 signaling is well characterized, the role of the structurally different cyanobacterial lipid A in TLR4 signaling is not well understood. The uncontrolled inflammatory response of TLR4 leads to autoimmune diseases such as sepsis, and thus the less virulent marine cyanobacterial LPS molecules can be effective to inhibit TLR4 signaling. This review highlights the structural comparison of LPS molecules from marine cyanobacteria and Gram-negative bacteria. We discuss the potential use of marine cyanobacterial LPS as a TLR4 antagonist, and the effects of cyanobacterial LPS on humans and marine organisms.

## 1. Introduction

Among the Gram-negative photosynthetic prokaryotes, cyanobacteria constitute a large group that is diverse in physiology, metabolism, and morphology [1]. Cyanobacterium is heterotrophic in nature, and rapidly grows in various habitats including terrestrial, fresh water, and marine ecosystems [1]. Cyanobacteria also grow under extreme conditions and are found in Antarctic lakes and both saline and hot springs. They dominate phytoplankton and, under favorable conditions, are involved in intense bloom formation in surface water with lower levels of dissolved oxygen, because it is enriched with minerals and organic nutrients [2]. Besides fixing nitrogen, cyanobacteria produce a diverse array of secondary metabolites and some of them are toxic in nature. Marine cyanobacteria may have evolved to produce a range of bioactive natural products and secondary metabolites as a defense strategy against herbivory and environmental stress factors present in a marine environment [3]. They show diverse bioactivities against a variety of pathogens and act as tumor- and immunosuppressants [4]. All types of cyanobacteria produce various toxins which are unique and can be pharmacologically active, such as Lipopolysaccharide (LPS) [5]. Based on their chemical structure and their effects on organs, these toxins can be classified into cytotoxins, neurotoxins, dermatotoxins, hepatotoxins, or irritant toxins [6,7]. Even though cyanobacterial LPSs possess clinical benefits, their properties are poorly characterized in comparison to other heterotrophic bacteria.

To date, different methods have been employed to analyze the structure of LPSs, including nuclear magnetic resonance (NMR) spectroscopy, mass spectrometry (MS), gas chromatography, and matrix-assisted laser desorption/ionization (MALDI)-MS [8]. These techniques are based on the following major steps: extraction, refinement and fragmentation [8].

The Gram-negative cell envelope has two membranes: a cytoplasmic membrane and an outer membrane. The endotoxic LPS molecules present in the outer membrane cover up to 3/4th of the total cell surface [9]. The cell envelope of cyanobacterial species shows a large overall resemblance with that of Gram-negative bacteria, but the peptidoglycan layer in the outer membrane is significantly thicker, comparable to that of Gram-positive bacteria [10]. The LPS molecules in marine Gram-negative bacteria as well as marine cyanobacteria, contribute greatly to the organism’s structural assembly and provide protection from antimicrobial compounds [11,12]. Many species of Gram-negative bacteria depend on LPS molecules for their survival as they also position cell membrane porins, which enable passage of ions and molecules [9].

In general, LPS consists of three essential structural parts: (1) a glycan with an *O*-specific polysaccharide, which is attached to (2) a glycolipid anchor lipid A, through (3) a connecting polysaccharide Core region [13]. LPS molecules from most Gram-negative and a few cyanobacterial species elicit a strong immune response. When a pathogen infects the host, TLR4, which is present on the surface of various cells, including neutrophils, monocytes, and macrophages, recognizes and binds LPS, and subsequently forms a complex with a small protein called MD-2 which activates downstream signaling [14]. However, an uncontrolled response of TLR4 signaling against LPS endotoxin can result in the inflammatory disease called sepsis. Sepsis is a severe condition leading to high fever and kidney or lung failure, and has a mortality rate of approximately 30 percent [13,15]. Like the terrestrial Gram-negative LPS, cyanobacterial LPS can be toxic to humans by causing allergy, or respiratory and skin diseases [16]. However, LPS molecules from marine Gram-negative and cyanobacteria are known to be less toxic to their host due to structural differences in comparison to common LPS [17]. Moreover, the lipid A portion of less toxic cyanobacterial LPS is structurally similar to a well-known TLR4 antagonist called lipid IVa [12]. Further studies may help to prevent anti-inflammatory diseases through cyanobacterial LPS.

## 2. Gram-Negative LPSs

**Figure 1:**
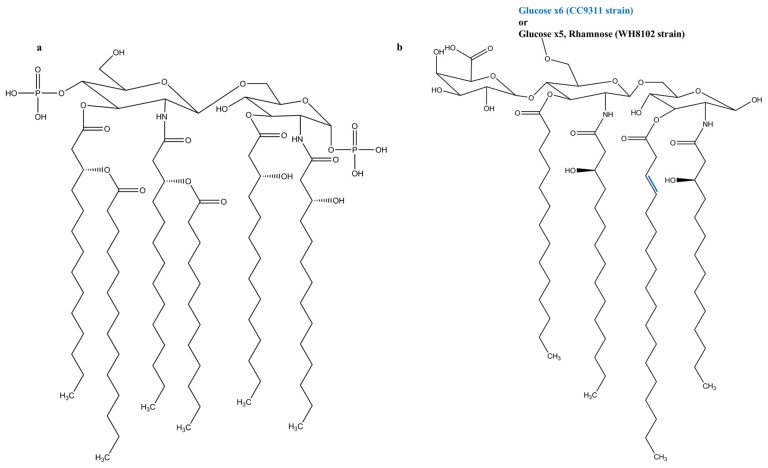
Lipid A Structures of (**a**) *E. coli*, a well-known TLR4 agonist; and (**b**) *Synechococcus* WH8102 and CC9311 strains.

The three regions of LPS are differentiated from one another based on their structure and function. The outermost *O*-specific polysaccharide chain has a polymerized repetitive unit that may contain around eight different sugars, usually C6 sugars, depending on the species and strain [18]. The *O*-chain contributes to antigenicity and is usually species-specific. The core oligosaccharide has about 10 sugars and can be divided into an inner and outer region. The inner region is highly conserved and contains the unusual sugars heptose and 3-deoxy-d-*manno*-octulosonic acid (also known as keto-deoxyoctulosonate (KDO)), whereas the outer region contains common sugars such as hexoses or hexosamines [18]. The innermost lipid A commonly has a bi-phosphorylated β-1,6-glucosamine disaccharide backbone connecting acyl chains through ester or amide linkage, anchoring the LPS molecule in the external membrane (Figure 1a) [13]. Some anionic groups, such as phosphates, are present on the lipid A and inner core regions [8]. The structure of lipid A is extremely conserved, whereas the core region is variable, and the *O*-specific polysaccharide part is highly variable [19]. There are two main types of LPSs depending on the structural composition: smooth-type and rough-type LPS. The former is the complete form of LPS with all three portions, while the latter lacks the *O*-specific polysaccharide portion [11]. While lipid A provides LPS with immunological and endotoxic properties, the role of the saccharide portion of LPS is also substantial [18].

## 3. Cyanobacterial LPSs

A few structures of cyanobacterial LPS have been reported and are discussed below per species. The quantity of key constituents in LPS of several cyanobacterial species are given in Table 1.

*Synechococcus* [12]: The marine cyanobacterial LPS molecules of *Synechococcus* strains WH8102 and CC9311 have neither heptose nor KDO. They are replaced by 4-linked glucose as a major saccharide constituent, and to a lesser extent by glucosamine and galacturonic acid. The lipid A portion contains odd-chain hydroxylated fatty acids, no phosphates, and comprises a single galacturonic acid (Figure 1b). This suggests that the LPSs of cyanobacteria vary from those of Gram-negative bacteria. The strains WH8102 and CC9311 have an α1,4-linked glucose chain, but while WH8102 has a single rhamnose, the core region of CC9311 only consists of glucose. The lack of tetraacyldisaccharide 4′ kinase in WH8102 and CC9311 indicates that the lipid A region is not phosphorylated.

*Microcystis aeruginosa*: LPSs from *M. aeruginosa* contain a large quantity of KDO, glucose, 3-deoxy sugars, glucosamine, fatty acids, fatty acid esters, hexoses, and phosphates [20]. Even though the complete chemical structure of *M. aeruginosa* LPS has not been obtained, its sugars were identified colorimetrically as dOclA, glucose, 3-deoxy sugars, and glucosamine [20]. Another study shows that the LPSs in two strains of *M. aeruginosa*, PCC 7806 and UV-017, contain glucose, mannose, xylose, galactose, fucose, and rhamnose, whereas KDO and heptoses were absent [21]. A third and recent study on the *O*-chain of *M. aeruginosa* confirms that it has the common neutral sugars glucose, rhamnose, xylose, mannose, and galactose, with glucose being the most abundant at 66% [22].

*Anacystis nidulans*: The lipid portion of *A. nidulans* LPS consists of a series of long fatty acyl chains including β-hydroxy-myristic acid. The carbohydrate region comprises mannose, glucose, galactose, fucose, rhamnose, 2-keto-3-deoxy octonic acid, glucosamine, and a second aminosugar, which is believed to be 2-amino-2-deoxy-heptose (with d-gluco configuration on C3–C7) [23]. *A. nidulans* LPS is composed of KDO and β-hydroxymyristic acid and is thus similar to Gram-negative LPS. In contrast, it lacks heptose, has low levels of phosphate, and has relatively little glucosamine in its lipid moiety [24].

*Agmenellum quadruplicatum* [25]: The presence of polar and non-polar regions in the LPS of *A. quadruplicatum* resembles the composition of other known LPSs. However, the presence of xylose in the polar region, unusual pentose sugars in the *O*-antigen region, and lack of galactose make *A. quadruplicatum* LPS unique. The presence of components such as rhamnose and mannose, and the absence of heptoses are in line with other known cyanobacterial LPS structures. *A. quadruplicatum* and *A. nidulans* have behenic acid in their LPS, and also have β-hydroxy fatty acids that are similar to the Gram-negative bacterial lipid A portion.

*Schizothrix calcicola* [26]: LPS from *S. calcicola* contains neutral sugars such as glucose, galactose, mannose, xylose, and rhamnose, and only the glucosamine amino sugar. KDO and heptose were absent like in a few other cyanobacterial species and *Bordetella* Gram-negative species. The lipid A portion contains β-hydroxylauric, myristic, pentadecanoic, palmitic, β-hydroxypalmitic, stearic, oleic, and linoleic acids.

*Anabaena* spp.: In addition to common core sugars and xylose, *Anabaena variabilis* has lacofriose, *Anabaena flos-aquae* has fucose, and *Anabaena cylindrica* has 3,6-dideoxyhexose [27]. Cyanobacterial LPS contains high amounts of oleic, palmitoleic, linoleic, and occasionally linolenic acids. The absence of the common Gram-negative LPS core components KDO and heptose, and the lack of phosphorus and glucosamine in the lipid A region differentiate the cyanobacterial LPS [27]. When compared to the lipid A portion of *Anabaena flos-aquae* UTEX 1444, *Anabaena variabilis*, lacks the palmitic acid, 3-β-hydroxy fatty acids, and 10 long chain saturated and unsaturated fatty acids.

*Spirulina platensis*: LPS from *S. platensis* has unsaturated fatty acids, 3-hydroxy myristate, and the carbohydrates, hexose, heptose, octulosonic acid and glucosamine [28]. The entire carbohydrate and fatty acid content represents almost half of the total LPS. Sugar investigation shows the presence of KDO, glucose, rhamnose, fucose, ribose, xylose, mannose, galactose, inositol, d-glycerol-d-*manno*-heptose, d-glycero-l-*manno*-heptose, and 3- or 4-*O*-methyl hexose. Lipid investigation found the occurrence of digalactosyl diacylglycerol and phosphatidyl diacylglycerol. Glycerol was also found in the hydrolysate. Glucosamine was the only amino sugar detected. Minor quantities of 3-OH-C_16_, were also detected. Lyso-forms of digalactosyl diacyl-glycerol, and phosphatidyl diacyl glycerol were not previously identified. The presence of the bulky portion of C_18_ polyunsaturated fatty acids is an uncommon feature for prokaryotes.

*Oscillatoria planktothrix* FP1: Similar to *Synechococcus* LPS, *Oscillatoria planktothrix* FP1 LPS is lack of KDO, heptose and phosphate, and the glucosamine disaccharidic backbone consists of hydroxylated and non-hydroxylated fatty acids [29]. At C4 position of distal glucosamine, *Synechococcus* LPS molecules have galactouronic acid and Gram-negative bacteria has phosphate group, respectively. Moreover, at C6 position the enterobacterial LPS has KDO but a chain of six 4-substituted glucose is present in *Synechococcus* LPS. In contrast to both the structures, galacturonic acid is present in LPS of *Oscillatoria planktothrix* FP1 at C6 position and is the main component in the core region providing negative charge. On the contrary, *Synechococcus* LPS has neutral residues such as glucose and rhamnose. The high molecular mass of *Oscillatoria planktothrix* FP1 LPS *O*-chain containing 3-substituted α-l-rhamnose residues and constitutes 3/4th of the glycosyl composition of LPS.

**Table 1:** The chemical composition of major components in cyanobacterial LPS. NA: Information not available.

Cyanobacterial Species	Carbohydrates (%)	Phosphorus (%)	KDO (%)	Proteins (%)	Fatty Acids (%)	References
*Schizothrix calcicola*	63	<0.1	Absent	7.8	8	[26]
*Phormidium* spp.	60	<1	0.5	7.20	NA	[30]
*Agmenellum quadruplicatum*	59.5	2.9	0.13	0.13	15.1	[25]
*Anabaena variabilis*	80.3	0.03	Absent	8.4	10.7	[27]
*Spirulina platensis*	31.6	0.6	NA	0.6	14.3	[28]
*Anacystis nidulans*	60	0.03	1.5	NA	12.4	[23,24]
*Microcystis aeruginosa*	36.0	0.7	Absent	0.4	18.2	[20,21]
*Anabaena flos-aquae*	65	Absent	12.5	NA	NA	[27]

## 4. Role of TLRs in Sepsis

The cases of severe sepsis and subsequent mortality are increasing globally [31]. In addition, approximately 1/3rd of its survivors develop severe functional limitations [32]. The uncontrolled host response to infection leads to sepsis, and caused by pattern recognition receptors including the well-studied TLRs [31]. As the first line of host defense during infection, TLRs recognize pathogen-associated molecular patterns (PAMPs) and damage associated molecular patterns (DAMP) that are microbial and indigenous molecules, respectively [33]. Vertebrate TLRs are evolutionarily grouped into six subfamilies: TLR1/2/6/10, TLR3, TLR4, TLR5, TLR7/8/9, and TLR11/12/13/21/22/23 [34]. Each member of the TLR family recognizes PAMPs of different kinds from a wide range of microbes to initiate downstream signaling [35]. Among TLRs, TLR4 is a key member in sensing LPS of Gram-negative bacteria to evade pathogens [36]. Moreover, Gram-negative LPS such as *Helicobacter pylori* LPS is recognized by TLR2 in addition to its main ligands lipopeptides, lipoproteins, and glycosylphosphatidylinositols [31,37]. Both TLR4 and TLR2 can also activate signaling by recognizing the same DAMP molecule HMGB1 [31]. TLR4 can induce the production of inflammatory cytokines via myeloid differentiation primary-response protein 88 (MyD88)-dependent and TIR-domain-containing adaptor protein inducing Interferon (IFN)-β (TRIF)-dependent pathways, whereas TLR2 undergoes only MyD88-dependent pathway [38]. TLRs form either homodimer or heterodimer to accommodate the ligand and initiate TLR signaling. TLR4 recognizes its agonists such as LPS in combination with accessory protein MD-2, while TLR2 forms heterodimer with either TLR1 or TLR6 [36]. However, the uncontrolled response after LPS recognition by TLRs results in sepsis and thus inhibiting TLR4 and TLR2 mediated signaling is an effective therapy for sepsis [31].

Among several approaches including the blockade of adapter molecules involved in TLR signaling, preventing LPS from binding to the host TLR looks most promising. Currently, no effective drug is available for sepsis despite a lot of research efforts in the recent past. The previously approved drug recombinant human activated protein C was withdrawn from the market due to its failure to control the death rate in patients between ~1 and 3 months [32]. Even though several TLRs are known to cause sepsis, the vast structural and functional information make TLR4 the most attractive target and TLR2 the next favorite for anti-sepsis treatment.

## 5. LPSs and TLR4 Signaling

**Figure 2:**
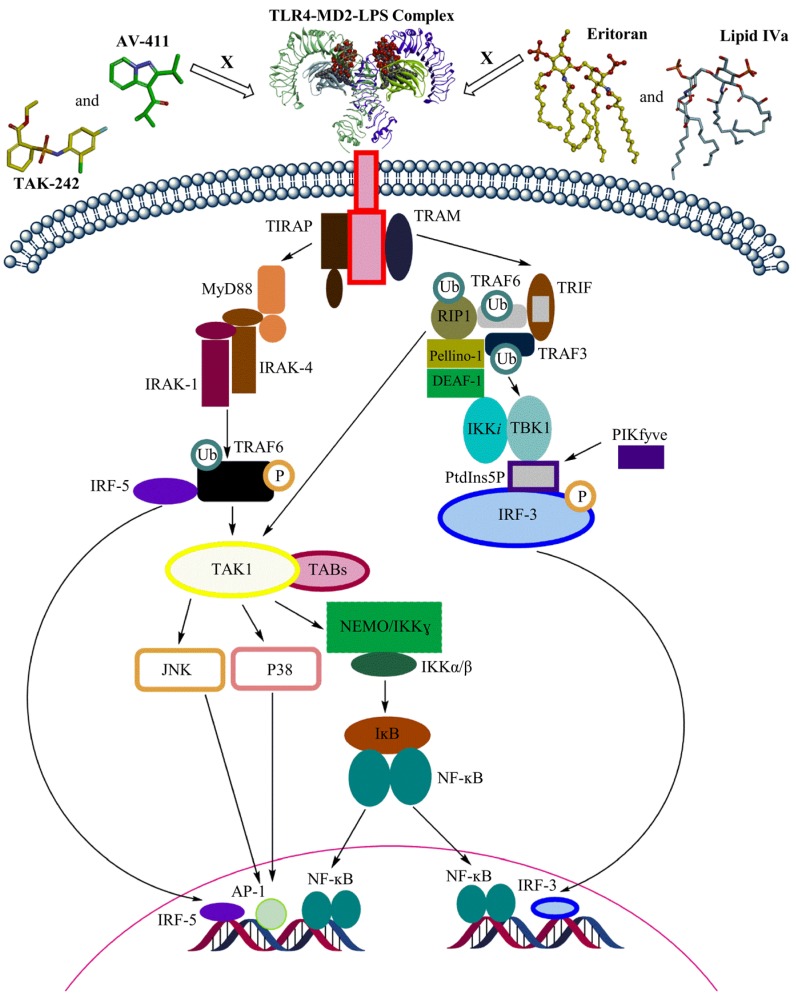
Overview of TLR4 signaling through MyD88-dependent and TRIF-dependent pathways.

The LPS-triggered TLR4 immune response plays a significant role in innate and adaptive immunity and its activation is determined by the lipid A portion [39]. LPS binding to the ectodomain (ECD) of TLR4 induces the formation of a TLR4-MD2-LPS complex, which will bring the Toll-IL-1 receptor (TIR) domains of TLR4 closer to each other to facilitate downstream signaling (Figure 2) [40]. TLR4 signaling consists of two downstream pathways: the MyD88 pathway, or the TRIF pathway [38]. In the MyD88 pathway, TIRAP binds to the TIR domain of TLR4 and recruits MyD88 to initiate the canonical pathway [38]. Successively, molecules including interleukin-1-receptor-associated kinase family members activate tumor-necrosis-factor receptor-associated factor (TRAF) 6, which leads to the subsequent activation of IFN regulatory factor (IRF) 5 and transforming-growth factor-β-activated kinase (TAK) 1 complex. The TAK1 complex will activate the IκB kinase (IKK) complex and the initiation of transcription of inflammatory genes. TAK1 complex activation will also activate MAPKs, which leads to JNK and p38 signaling and subsequent AP-1 activation [35,41]. On the other hand, in the TRIF dependent pathway, TRAM binds to the TIR domain of TLR4, which leads to the sequential recruitment of TRIF, TRAF6, and TRAF3 [33]. TRAF6 employs RIP-1 to activate the TAK1 complex and to regulate ubiquitination by Pellino-1. Pellino-1 controls IRF3 stimulation by adhering to DEAF-1 [42]. TRAF3 employs TRAF-family-member-associated NF-κB activator binding kinase (TBK) 1 and IKKi for IRF3 phosphorylation. PtdIns5P from PIKfyve assists complex formation between TBK1 and IRF3 [42]. Both TLR4 signaling pathways are vital to initiate the first line of defense against the invading pathogens [43].

However, excessive activation of TLR4 can lead to autoimmune disorders and inflammatory diseases [44]. TLR4 blockage can be achieved through various inhibitors which can act on the molecules essential for downstream signaling or even bind to the ECD and prevent TLR dimerization [39]. Eritoran and lipid IVa are well-known antagonists of human TLR4 that act on the ECD. Lipid IVa is a derivative of the agonist LPS obtained by reducing the number of fatty acid acyl chains [45]. In contrast to its antagonist activity towards the human TLR4-MD-2 complex, lipid IVa shows agonistic activity in the mouse TLR4-MD-2 complex [36]. Moreover, small-molecule TLR4 antagonists such as TAK-242 and AV-411 are available, but none of them have been approved as a drug [44,46]. The possible role of LPS from marine Gram-negative bacteria as TLR4 antagonist has been recently reviewed [46]. In addition, there are evidences that cyanobacterial LPS can antagonize Gram-negative LPS-induced immune responses. The main structural differences in cyanobacterial LPS are the length of the fatty acid chain in the lipid A region and the lack of heptose, phosphates, and KDO in the oligosaccharide core region.

## 6. Cyanobacterial LPSs in TLR4 Signaling

Cyanobacteria produce a wide range of biologically active compounds, including their pharmacologically important LPS molecules. Nevertheless, they are the least studied species of marine bacteria. The LPS molecules can be extracted from a wide variety of cyanobacteria, but not all of them can act as an effective TLR4 antagonist, and several may not even show any activity towards TLR4. However, cyanobacterial LPS is structurally similar to the common LPS structure and about an order of magnitude less toxic than the Gram-negative LPS. Hence, they can be TLR4 antagonists and a few studies supporting this claim are discussed here. The LPS-like molecule CyP obtained from the cyanobacterium *Oscillatoria planktothrix* FP1 inhibited *E. coli* LPS-induced TLR4 signaling in human dendritic cells (DCs) [47]. Moreover, CyP inhibited both the MyD88 dependent and independent TLR4 pathways and did not show any toxicity towards human or mouse cells [47]. Blockade of both the possible pathways for TLR4 activation by CyP confirms its accommodation in ECD of TLR4 and competition with LPS to bind in the active site. In addition, CyP also protected mice from endotoxic shock induced by Gram-negative *Salmonella abortus* LPS [47]. The CyP molecule was also tested on porcine whole blood and it abrogated *E. coli* LPS-induced TLR4 signaling [48]. It therefore suggests that CyP is not species-specific in antagonizing TLR4. During an *in vitro* experiments with human DCs and TLR4-transfected Jurkat cells, the CyP molecule prevented *Neisseria meningitidis* LPS from binding to TLR4, lowering inflammatory response and preventing septicemia [49]. However, CyP was effective only when treated to blood within 1 h of treatment with meningococci or meningococcal components. In an *in vitro* study using meningitis model, CyP was co-treated with benzylpenicillin in cell monocultures and macrophage-meningioma cell co-cultures and found to inhibit the *N. meningitidis* LPS-induced production of cytokines IL-6, IL-8 and IL-1B [50]. Cyp alone was effective in inhibiting the secretion of monocyte chemotactic protein (MCP)-1 and RANTES, but failed to do so in case of TNF-α [50]. In an *in vitro*/*in vivo* study, VB3323, a 95% purified Cyp from *Oscillatoria planktothrix* sp., was neuroprotective by inhibiting TLR4-activatd cytokines that are responsible for motor neuron degeneration [51]. Its level of TLR4 antagonism is close to the well-known Gram-negative *Rhodobacter sphaeroides* LPS. Contrasting to TLR4 signal hindering activity of CyP, 20 g/mL of its concentration exhibited TLR4 agonistic activity in whole blood. This is suspected to be caused by glycolipid that is similar to LPS used during preparation, but may have other reasons too for its induction of IL-8 and MCP-1 [47].

The level of toxicity of LPSs is dependent on the number and the organization of their fatty acid chains, their phosphorylation, and the presence of charged groups [9,39]. The structural variations of lipid A among the species and strains are due to genetic dissimilarities or growth conditions [52]. The length of the fatty acid chains, the number of fatty acid chains, and the symmetric or asymmetric acylation patterns may all be different [9,52]. In addition to these parameters, the secondary fatty acid chains may also present either as saturated or unsaturated acyl chains [9]. The absence of phosphate groups in the Lipid A region of Gram-negative bacteria *Francisella tularensis* is believed to be the reason for its poor immune response but proves they are not essential for immune activation [53,54]. Based on the differences in the lipid A region, which carries the endotoxic properties in LPS, the cyanobacterial LPSs are found to be less toxic than Gram-negative bacteria and thus are more suitable to control TLR4 signaling.

The lipid A structure or oligosaccharide core possibly cause the less endotoxin-like activity of the cyanobacterial LPS. Empirical data indicate that the number of lipid chains in the lipid A portion of LPS mainly dictates the type of TLR4-dependent immune response. Lipid A with six chains is a potent TLR4 agonist, but Lipid A with five or seven chains is 100 times less effective [39]. The effective TLR4 antagonists Eritoran and lipid IVa consist of only four lipid chains, and they differ in the number of carbon atoms and double bonds in their lipid chains [36]. Similarly, the recently characterized *Synechococcus* LPS [12] has the same number of lipid chains which shows the possibility that most of the cyanobacterial LPSs may contain four lipid chains and may be accommodated in the MD-2 cavity and prevent dimerization with TLR4. A potent TLR4 antagonist has also been reported with five fatty acid chains from two *Rhodobacter* species [55]. Further studies on cyanobacterial LPS molecules are needed to establish their potential role as TLR4 antagonists.

## 7. Biological Activity of Cyanobacterial LPSs

A few details on biological effects of cyanobacterial LPS have been reported and are explained below per species.

LPS from *Anabaena flos-aquae* was injected intraperitoneally into mice and found to be non-toxic, whereas LPSs from *O. brevis* and *Anabaena cylindrica* are active in mice but 90% less than *Salmonella* sp. [27]. LPS from *S. calcicola* was not toxic to mice when injected intraperitoneally but caused Limulus amebocyte lysate (LAL) gelation and showed a positive response in Schwartzman reaction [26]. *Agnemellum* LPS is positive in local Schwartzman reaction and LAL reaction but less toxic than *Salmonella* LPS [25]. The LPS of *M. aeruginosa* showed less pyrogenecity in human blood compared to *E. coli* LPS [56]. The toxic activity in mice and LAL gelation activity of *M. aeruginosa* LPS from the isolates 006 and NRC-1 were positive, but slightly less active than *S. abortus equi* LPS. The observed activities were clearly lipid A dependent [20]. LAL gelation of *Synechococcus* WH8102 LPS was negative [12], and *Synechococcus* PCC6910 and 6312 exhibit a 1000 times lower toxicity compared to *Salmonella*. The pyrogenecity of *A. nidulans* LPS was 10 times lower than those induced by *E. coli* LPS in rabbits, and no toxic effects were observed on mice [23]. Another study shows that *A. nidulans* LPS is about 800 times less toxic than *S. typhimurium* in adrenal-ectomized mice [24]. *Spirulina platensis* showed 10% of the toxic effects of *S. abortus equi* in a LAL gelation assay.

## 8. Effects of Cyanobacterial LPSs on Humans

LPS molecules are also called “endotoxins” or “irritant toxins” because of their dermatotoxic and inflammatory properties. Their participation in toxic shock syndrome may increase the hepatic damage triggered by hepatotoxins [57]. Some descriptions of allergies due to cyanobacteria have also been published. Cyanobacterial LPS is involved in various human diseases including skin diseases, gastro-intestinal issues, respiratory diseases, fever, allergy, and headache [58]. These are indirect effects caused by LPS and lead to innate immune responses.

The lipid A moiety is believed to be responsible for the toxic effects in different types of Gram-negative bacteria; it can be variable and sometimes even completely inactive. A few tests have indicated that cyanobacteria can induce skin sensitivity which may be caused by LPS molecules [57]. An outbreak of gastro-enteritis is suspected to have been caused by cyanobacterial LPS [17]. Cyanobacterial LPSs can cause strong allergic reactions, and skin and eye irritations [59]. They also induce the symptoms usual for influenza: rigors, headaches, queasiness, arthralgia, somnolence, slight memory loss, and diarrhea [59]. While cyanobacterial LPS molecules are found to be 10 times less toxic than other bacterial variants, gastrointestinal illness, respiratory sickness, and fever due to these toxins do exist [23].

## 9. Effects of Cyanobacterial LPSs on Marine Organisms

The activities of microsomal (m) and soluble (s) glutathione *S*-transferases (GST) were analyzed in embryos of the zebra fish, *Danio rerio*, that were treated from the time of fertilization to the prim six embryo stage with LPSs from several species [60]. In addition, the study examined how the activity was affected by co-treatment of LPS and microcystin-LR (MC-LR). sGST catalyzes the enzymatic conjugation of MC-LR to glutathione, which is common in aquatic organisms. Embryos treated with LPS molecules from *S. typhimurium*, *E. coli*, *Microcystis* CYA 43, and natural cyanobacterial blooms of *Microcystis* and *Gloeotrichia* displayed the lowest activities of mGST. The sGST activity was also reduced after the treatment with LPSs from those species, except with *S. typhimurium* or *E. coli* LPS. During the co-treatment with MC-LR and cyanobacterial LPS in the embryos, both m- and sGSTs showed reduced activity. *In vitro* preparations of GST from both adult and prim six embryo *D. rerio* showed lower activities after treatment with *Gloeotrichia* bloom LPSs. These results indicate that the cyanobacterial LPS molecules reduce the ability of *D. rerio* to detoxify microcystins.

In another but similar study, the effects of LPSs from *Microcystis* on several detoxifying enzymes/pathways including GST, GPx, GR, SOD, and CAT, were compared with those caused by LPSs from *E. coli* [61]. The LPS molecules from *Microcystis* showed a reduction of GST activity in *D. rerio* embryos, with a moderate increase in GPx and no effect on reciprocal GR activity.

A recent study on the effects of LPSs from *Lyngbya* spp. and *M. aeruginosa* shows that cadmium toxicity was increased after co-treatment of *D. rerio* embryos with cadmium and cyanobacterial LPS molecules [62]. In addition, GST activity was increased in the embryos during co-exposure, which proves that cyanobacterial LPSs increase cadmium toxicity regardless of the underlying mechanisms.

In a toxicological study, rainbow trout (*Oncorhynchus mykiss*) was co-treated with cyanobacterial LPSs and complete or fragmented microcystins from *Microcystis* (PCC 7813) [63]. An increase in liver weight and water quantity was observed in the gut upon co-treatment, but not upon microcystins treatment alone. This shows that cyanobacterial LPSs affect the liver detoxification system, similar to Gram-negative bacteria.

## References

[1] Berg K.A., Lyra C., Sivonen K., Paulin L., Suomalainen S., Tuomi P., Rapala J. (2009). High diversity of cultivable heterotrophic bacteria in association with cyanobacterial water blooms. ISME J..

[2] Sotero-Santos R.B., Silva C.R., Verani N.F., Nonaka K.O., Rocha O. (2006). Toxicity of a cyanobacteria bloom in barra bonita reservoir (middle tiete river, Sao Paulo, Brazil). Ecotoxicol. Environ. Saf..

[3] Tan L.T. (2007). Bioactive natural products from marine cyanobacteria for drug discovery. Phytochemistry.

[4] Dixit R.B., Suseela M.R. (2013). Cyanobacteria: Potential candidates for drug discovery. Antonie Van Leeuwenhoek.

[5] Chorus I., Falconer I.R., Salas H.J., Bartram J. (2000). Health risks caused by freshwater cyanobacteria in recreational waters. J. Toxicol. Environ. Health B Crit. Rev..

[6] Wang L., Liang X.F., Liao W.Q., Lei L.M., Han B.P. (2006). Structural and functional characterization of microcystin detoxification-related liver genes in a phytoplanktivorous fish, nile tilapia (*Oreochromis niloticus*). Comp. Biochem. Physiol. C Toxicol. Pharmacol..

[7] Wiegand C., Pflugmacher S. (2005). Ecotoxicological effects of selected cyanobacterial secondary metabolites: A short review. Toxicol. Appl. Pharmacol..

[8] Anwar M.A., Choi S. (2014). Gram-negative marine bacteria: Structural features of lipopolysaccharides and their relevance for economically important diseases. Mar. Drugs.

[9] Molinaro A., Holst O., Di Lorenzo F., Callaghan M., Nurisso A., D’Errico G., Zamyatina A., Peri F., Berisio R., Jerala R. (2015). Chemistry of lipid a: At the heart of innate immunity. Chemistry.

[10] Hoiczyk E., Hansel A. (2000). Cyanobacterial cell walls: News from an unusual prokaryotic envelope. J. Bacteriol..

[11] Leone S., Silipo A., Nazarenko E.L., Lanzetta R., Parrilli M., Molinaro A. (2007). Molecular structure of endotoxins from Gram-negative marine bacteria: An update. Mar. Drugs.

[12] Snyder D.S., Brahamsha B., Azadi P., Palenik B. (2009). Structure of compositionally simple lipopolysaccharide from marine *synechococcus*. J. Bacteriol..

[13] Caroff M., Karibian D. (2003). Structure of bacterial lipopolysaccharides. Carbohydr. Res..

[14] Wang X., Quinn P.J. (2010). Lipopolysaccharide: Biosynthetic pathway and structure modification. Prog. Lipid Res..

[15] Sperandeo P., Deho G., Polissi A. (2009). The lipopolysaccharide transport system of Gram-negative bacteria. Biochim. Biophys. Acta.

[16] Stewart I., Schluter P.J., Shaw G.R. (2006). Cyanobacterial lipopolysaccharides and human health—A review. Environ. Health.

[17] Hunter P.R. (1998). Cyanobacterial toxins and human health. Symp. Ser. Soc. Appl. Microbiol..

[18] Wilkinson S.G. (1996). Bacterial lipopolysaccharides—Themes and variations. Prog. Lipid Res..

[19] Moran A.P., Mobley H.L.T., Mendz G.L., Hazell S.L. (2001). Molecular structure, biosynthesis, and pathogenic roles of lipopolysaccharides. Helicobacter pylori: Physiology and Genetics.

[20] Raziuddin S., Siegelman H.W., Tornabene T.G. (1983). Lipopolysaccharides of the cyanobacterium *Microcystis aeruginosa*. Eur. J. Biochem..

[21] Martin C., Codd G.A., Siegelman H.W., Weckesser J. (1989). Lipopolysaccharides and polysaccharides of the cell envelope of toxic *Microcystis aeruginosa* strains. Arch. Microbiol..

[22] Fujii M., Sato Y., Ito H., Masago Y., Omura T. (2012). Monosaccharide composition of the outer membrane lipopolysaccharide and *O*-chain from the freshwater cyanobacterium *Microcystis aeruginosa* NIES-87. J. Appl. Microbiol..

[23] Weise G., Drews G., Jann B., Jann K. (1970). Identification and analysis of a lipopolysaccharide in cell walls of the blue-green alga *Anacystis nidulans*. Arch. Mikrobiol..

[24] Katz A., Weckesser J., Drews G., Mayer H. (1977). Chemical and biological studies on the lipopolysaccharide (*O*-antigen) of *Anacystis nidulans*. Arch. Microbiol..

[25] Buttke T.M., Ingram L.O. (1975). Comparison of lipopolysaccharides from *Agmenellum quadruplicatum* to *Escherichia coli* and *Salmonella typhimurium* by using thin-layer chromatography. J. Bacteriol..

[26] Keleti G., Sykora J.L., Lippy E.C., Shapiro M.A. (1979). Composition and biological properties of lipopolysaccharides isolated from *Schizothrix calcicola* (Ag.) Gomont (Cyanobacteria). Appl. Environ. Microbiol..

[27] Keleti G., Sykora J.L. (1982). Production and properties of cyanobacterial endotoxins. Appl. Environ. Microbiol..

[28] Tornabene T., Bourne T., Raziuddin S., Ben-Amotz A. (1985). Lipid and lipopolysaccharide constituents of cyanobacterium *Spirulina platensis* (Cyanophyceae, Nostocales). Mar. Ecol. Prog. Ser..

[29] Carillo S., Pieretti G., Bedini E., Parrilli M., Lanzetta R., Corsaro M.M. (2014). Structural investigation of the antagonist LPS from the cyanobacterium *Oscillatoria planktothrix* FP1. Carbohydr. Res..

[30] Mikheyskaya L.V., Ovodova R.G., Ovodov Y.S. (1977). Isolation and characterization of lipopolysaccharides from cell walls of blue-green algae of the genus *Phormidium*. J. Bacteriol..

[31] Ianaro A., Tersigni M., D’Acquisto F. (2009). New insight in LPS antagonist. Mini Rev. Med. Chem..

[32] Savva A., Roger T. (2013). Targeting Toll-like receptors: Promising therapeutic strategies for the management of sepsis-associated pathology and infectious diseases. Front. Immunol..

[33] Kumar H., Kawai T., Akira S. (2011). Pathogen recognition by the innate immune system. Int. Rev. Immunol..

[34] Roach J.C., Glusman G., Rowen L., Kaur A., Purcell M.K., Smith K.D., Hood L.E., Aderem A. (2005). The evolution of vertebrate Toll-like receptors. Proc. Natl. Acad. Sci. USA.

[35] Akira S., Uematsu S., Takeuchi O. (2006). Pathogen recognition and innate immunity. Cell.

[36] Botos I., Segal D.M., Davies D.R. (2011). The structural biology of Toll-like receptors. Structure.

[37] Durai P., Govindaraj R.G., Choi S. (2013). Structure and dynamic behavior of Toll-like receptor 2 subfamily triggered by malarial glycosylphosphatidylinositols of *Plasmodium falciparum*. FEBS J..

[38] Akira S., Takeda K. (2004). Toll-like receptor signalling. Nat. Rev. Immunol..

[39] Kang J.Y., Lee J.O. (2011). Structural biology of the Toll-like receptor family. Annu. Rev. Biochem..

[40] Park B.S., Song D.H., Kim H.M., Choi B.S., Lee H., Lee J.O. (2009). The structural basis of lipopolysaccharide recognition by the TLR4-MD-2 complex. Nature.

[41] Kawai T., Akira S. (2010). The role of pattern-recognition receptors in innate immunity: Update on Toll-like receptors. Nat. Immunol..

[42] Kawasaki T., Kawai T. (2014). Toll-like receptor signaling pathways. Front. Immunol..

[43] Kawai T., Akira S. (2006). TLR signaling. Cell Death Differ..

[44] Kondo T., Kawai T., Akira S. (2012). Dissecting negative regulation of Toll-like receptor signaling. Trends Immunol..

[45] Jin M.S., Lee J.O. (2008). Structures of the Toll-like receptor family and its ligand complexes. Immunity.

[46] Nijland R., Hofland T., van Strijp J.A. (2014). Recognition of LPS by TLR4: Potential for anti-inflammatory therapies. Mar. Drugs.

[47] Macagno A., Molteni M., Rinaldi A., Bertoni F., Lanzavecchia A., Rossetti C., Sallusto F. (2006). A cyanobacterial LPS antagonist prevents endotoxin shock and blocks sustained TLR4 stimulation required for cytokine expression. J. Exp. Med..

[48] Thorgersen E.B., Macagno A., Rossetti C., Mollnes T.E. (2008). Cyanobacterial LPS antagonist (CyP)—A novel and efficient inhibitor of Escherichia coli LPS-induced cytokine response in the pig. Mol. Immunol..

[49] Jemmett K., Macagno A., Molteni M., Heckels J.E., Rossetti C., Christodoulides M. (2008). A cyanobacterial lipopolysaccharide antagonist inhibits cytokine production induced by *Neisseria meningitidis* in a human whole-blood model of septicemia. Infect. Immun..

[50] Oliver R., Staples K.J., Heckels J., Rossetti C., Molteni M., Christodoulides M. (2012). Coadministration of the cyanobacterial lipopolysaccharide antagonist CyP with antibiotic inhibits cytokine production by an in vitro meningitis model infected with *Neisseria meningitidis*. J. Antimicrob. Chemother..

[51] De Paola M., Mariani A., Bigini P., Peviani M., Ferrara G., Molteni M., Gemma S., Veglianese P., Castellaneta V., Boldrin V. (2012). Neuroprotective effects of Toll-like receptor 4 antagonism in spinal cord cultures and in a mouse model of motor neuron degeneration. Mol. Med..

[52] Raetz C.R., Reynolds C.M., Trent M.S., Bishop R.E. (2007). Lipid a modification systems in Gram-negative bacteria. Annu. Rev. Biochem..

[53] Beasley A.S., Cotter R.J., Vogel S.N., Inzana T.J., Qureshi A.A., Qureshi N. (2012). A variety of novel lipid a structures obtained from *Francisella tularensis* live vaccine strain. Innate. Immun..

[54] Schilling B., McLendon M.K., Phillips N.J., Apicella M.A., Gibson B.W. (2007). Characterization of lipid a acylation patterns in *Francisella tularensis*, *Francisella novicida*, and *Francisella philomiragia* using multiple-stage mass spectrometry and matrix-assisted laser desorption/ionization on an intermediate vacuum source linear ion trap. Anal. Chem..

[55] Rose J.R., Christ W.J., Bristol J.R., Kawata T., Rossignol D.P. (1995). Agonistic and antagonistic activities of bacterially derived *Rhodobacter sphaeroides* lipid A: Comparison with activities of synthetic material of the proposed structure and analogs. Infect. Immun..

[56] Blahova L., Adamovsky O., Kubala L., Svihalkova Sindlerova L., Zounkova R., Blaha L. (2013). The isolation and characterization of lipopolysaccharides from *Microcystis aeruginosa*, a prominent toxic water bloom forming cyanobacteria. Toxicon.

[57] Zanchett G., Oliveira-Filho E.C. (2013). Cyanobacteria and cyanotoxins: From impacts on aquatic ecosystems and human health to anticarcinogenic effects. Toxins (Basel).

[58] Rapala J., Lahti K., Rasanen L.A., Esala A.L., Niemela S.I., Sivonen K. (2002). Endotoxins associated with cyanobacteria and their removal during drinking water treatment. Water Res..

[59] Jakubowska N., Szelag-Wasielewska E. (2015). Toxic picoplanktonic cyanobacteria—Review. Mar. Drugs.

[60] Best J.H., Pflugmacher S., Wiegand C., Eddy F.B., Metcalf J.S., Codd G.A. (2002). Effects of enteric bacterial and cyanobacterial lipopolysaccharides, and of microcystin-LR, on glutathione *S*-transferase activities in zebra fish (*Danio rerio*). Aquat. Toxicol..

[61] Jaja-Chimedza A., Gantar M., Mayer G.D., Gibbs P.D., Berry J.P. (2012). Effects of cyanobacterial lipopolysaccharides from microcystis on glutathione-based detoxification pathways in the zebrafish (*Danio rerio*) embryo. Toxins (Basel).

[62] Notch E.G., Miniutti D.M., Berry J.P., Mayer G.D. (2011). Cyanobacterial LPS potentiates cadmium toxicity in zebrafish (*Danio rerio*) embryos. Environ. Toxicol..

[63] Ferrao-Filho Ada S., Kozlowsky-Suzuki B. (2011). Cyanotoxins: Bioaccumulation and effects on aquatic animals. Mar. Drugs.

